# Associations between genes methylation, postnatal risk factors and psychiatric symptoms in a clinical sample of children and adolescents: Preliminar results from the remind longitudinal study

**DOI:** 10.1192/j.eurpsy.2021.347

**Published:** 2021-08-13

**Authors:** F. Villa, E. Rosi, S. Grazioli, M. Mauri, R. Giorda, P. Brambilla, C. Bonivento, M. Garzitto, M. Molteni, M. Nobile

**Affiliations:** 1 Developmental Psychopathology, Scientific Institute Eugenio Medea, Associazione La Nostra Famiglia, Bosisio Parini, Italy; 2 Molecular Biology Laboratory, Scientific Institute, IRCCS E. Medea, Bosisio Parini, Italy; 3 Department Of Pathophysiology And Transplantation, University of Milan, Milan, Italy; 4 Associazione La Nostra Famiglia, Scientific Institute, IRCCS E. Medea, Pasian di Prato, Italy; 5 Child And Adolescent Psychiatry Unit, Scientific Institute IRCCS ‘E. Medea?, Bosisio Parini, Italy

**Keywords:** methylation, epigenetics, postnatal risk factors, psychopathology trajectories

## Abstract

**Introduction:**

Epigenetics hypothesizes a crucial link between postnatal risk factors, individual response to stress, DNA methylation and psychiatric symptomatology changes during life.

**Objectives:**

We analyzed methylation within two gene exons: NR3C1 and SLC6A4, which are involved in responses to environmental stressors. We investigated the relationship between methylation, postnatal risk factors and psychopathology assessed by Child Behavior Checklist (CBCL) in our help-seeking sample evaluated in infancy (W1), preadolescence (W2) and adult life (W3).

**Methods:**

Postnatal risk factors data were collected at W1 in 205 clinical subjects (156 M, 49 F; age=9,13±1,95). The CBCL scores were collected at W1 and W2 (W2 age=14,52±2,12). Data regarding methylation were collected at W2. At W3 we are also collecting clinical scores. A Spearman correlation coefficient was calculated between methylation percentage and clinical data at W2. The externalizing and internalizing trajectories were evaluated through repeated measure ANOVA with postnatal risk factors (presence/absence) as between-groups factor.

**Results:**

Significant associations were found between methylation and internalizing and total clinical scores (Table 1). The rm-ANOVA results showed a significant interaction between the CBCL internalizing score and presence/absence of postnatal risk, with higher internalizing problems in subjects that were exposed to postnatal risk factors. This effect was significant at W2 but not at W1 (Figure 1).

**Figure:**
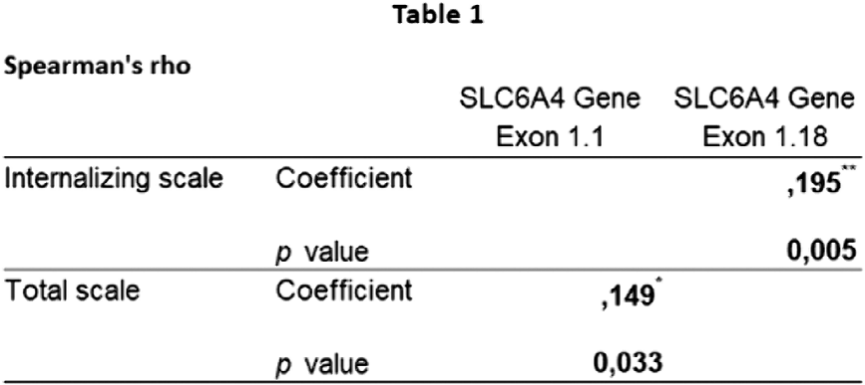

**Figure:**
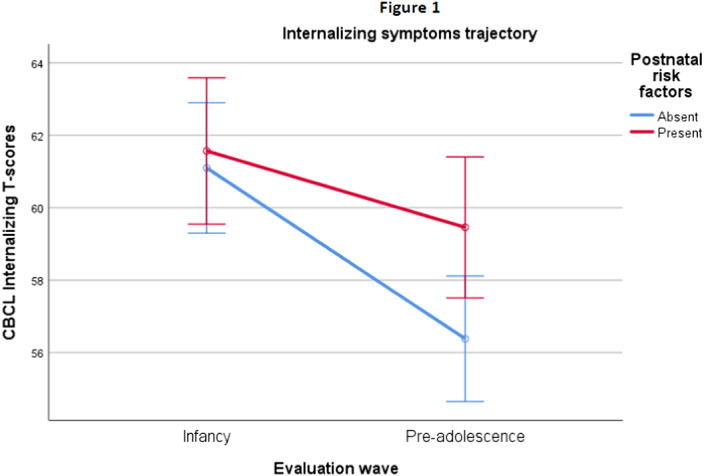

**Conclusions:**

Psychopathological symptoms trajectories could depend on epigenetics and early environmental risk factors. Further analyses will address a Linear Discriminant Analysis to proceed to a machine learning oriented approach.

**Disclosure:**

No significant relationships.

